# *Scorpio fuscus* venom as a promising anticancer agent against colorectal cancer

**DOI:** 10.1007/s10637-026-01598-z

**Published:** 2026-02-02

**Authors:** Serdar Karakurt, Cigdem Gokcek-Sarac, Sinan Kandir, Irem Mukaddes Bilgiseven, Ersen Aydın Yagmur, Hasan Ufuk Celebioglu

**Affiliations:** 1 Department of Biochemistry, Faculty of Science, Selcuk University, Konya, Türkiye; 2 Faculty of Medicine and Health Technology, BioMediTech Institute, University of Tampere, Tampere, Finland; 3 Department of Biomedical Engineering, Faculty of Engineering, Akdeniz University, Antalya, Türkiye; 4 Department of Physiology, Faculty of Veterinary Medicine, Cukurova University, Adana, Türkiye; 5 Alasehir Vocational High School, Manisa Celal Bayar University, Manisa, Türkiye; 6 Department of Biotechnology, Faculty of Science, Bartin University, Bartin, Türkiye

**Keywords:** *Scorpio fuscus*, Venom, Colorectal carcinoma, In vivo, Molecular mechanism, Signaling pathways

## Abstract

Colorectal cancer (CRC) remains a leading cause of cancer-related mortality worldwide, underscoring the urgent need for novel and effective therapeutic agents. Conventional treatments, including chemotherapy, radiotherapy, and surgery, are often associated with significant adverse effects, prompting the exploration of alternative therapeutic strategies. This study aimed to evaluate the anticancer effects of *Scorpio fuscus* venom (*SFV*) on human colorectal cancer models using integrated in vitro and in vivo approaches. *SFV* constituents were characterized using gel electrophoresis, followed by high-performance liquid chromatography and UV–visible spectrometry. Identified peptides were subjected to structural modeling and in silico docking analyses against selected proteins associated with colorectal cancer and apoptosis-related pathways. The cytotoxic effects of *SFV* were assessed in human CRC cell lines (DLD-1, HT-29, and CaCo-2) and a healthy colon epithelial cell line (CCD-18Co) using Alamar Blue assays after 48-h treatment, and half-maximal inhibitory concentration (IC₅₀) values were determined. *SFV* treatment resulted in a dose-dependent reduction in cancer cell viability, accompanied by decreased migratory capacity and colony formation ability. Apoptotic responses were further evaluated by flow cytometry and gene expression analyses, indicating modulation of apoptosis-associated genes. For in vivo validation, subcutaneous and orthotopic xenograft colon cancer models were established in mice. *SFV* administration led to reduced tumor growth compared with control groups. Immunohistochemical analyses revealed altered expression patterns of selected tumor-related markers in *SFV*-treated tumors. Gene expression profiling demonstrated ≥ twofold changes in 51 genes, including downregulation of BAK1 and TRAF3, and upregulation of BIRC2, BIRC3, BIRC6, CASP8, TNFRSF8, TNFRSF11, and BOK. Collectively, these findings indicate that *SFV* exerts significant antitumor effects in colorectal cancer models and support its potential as a promising anticancer agent, warranting further mechanistic and translational investigation.

## Introduction

Colorectal cancer (CRC) is the third most prevalent cancer worldwide and the second most lethal form of cancer [1]. According to Sawicki et al. (2021), the incidence of colon and rectal cancer is expected to increase by 71.5% and 60.0%, respectively, by 2035 [2]. Surgical excision is the primary treatment for resectable CRC, while chemotherapy, radiotherapy, and immunotherapy are the standard treatments for unresectable CRC. Nevertheless, these therapies have certain disadvantages, including high cytotoxicity and lack of specificity, which can result in secondary complications [3]. Although these therapies can be combined based on the extent and progression of the CRC, over half of the patients still relapse with multidrug-resistant CRC [4]. Despite significant progress in CRC screening, surgery, and adjuvant treatment, the death rate remains high, highlighting the urgent need for innovative therapies that increase the sensitivity of resistant tumors to chemotherapy [5].

Recent studies have revealed the role of numerous oncogenic factors, immune regulatory mechanisms, and adaptive survival pathways in the molecular complexity and heterogeneity of colorectal cancer [6, 7]. Changes in immune homeostasis and tumor-microenvironment interactions contribute to treatment resistance, while the abnormal expression of tumor-associated genes and non-coding RNAs has been reported to influence tumor progression, metastatic behavior, and patient prognosis [8, 9]. Furthermore, evidence continues to accumulate that dysregulated signaling networks, mitochondrial dynamics, autophagy, and cellular stress responses significantly influence the progression of colorectal cancer and its response to treatment [10, 11]. Collectively, these findings emphasize the urgent need for novel therapeutic strategies that target adaptive resistance mechanisms and improve the efficacy of existing treatments.

Biologically active compounds from animals and plants have been used to treat many diseases for years. The venoms of animals such as scorpions, snakes, and bees have been used for centuries in treating various diseases, including rheumatism and cancer. Scorpions, with a history dating back 430 million years, are among the oldest and highly toxic arachnids, classified under the phylum Arthropoda and the class Arachnida [12]. The genus *Scorpio* has been represented by only a single species, *Scorpio maurus* Linnaeus, 1758, containing 19 subspecies [13]. *Scorpio fuscus* was described initially with the protonym *Buthus (Heterometrus) palmatus fuscus* from Beirut (Lebanon) [14]. Scorpions are found on every continent except Antarctica. Among more than 2000 known species [15], around 1500 have venom, and perhaps 50 pose a significant danger to humans [16].

Aside from their taxonomy, scorpions are of biomedical interest due to their diverse venomous characteristics. Their venom comprises many components, including water, mucus, enzymes, proteins, small-molecule peptides, biogenic amines, polysaccharides, glycoproteins, and many unidentified molecules. Well-studied groups of these peptides include neurotoxins that target ion channels in the nervous system and potassium channel-blocking toxins, such as charybdotoxin, which block potassium channels and alter the excitability of neurons and muscles [17]. Scorpion venoms also contain metalloproteases, hyaluronidases, and phospholipases, which contribute to tissue degradation, cell membrane disruption, and the spread of venom throughout the prey’s body [18]. Moreover, antimicrobial peptides in scorpion venom exhibit activity against bacteria, fungi, and even cancer cells, highlighting their therapeutic potential [19].

This biochemical diversity makes scorpion venom a rich source for pharmacological research and drug development. Studies have demonstrated that scorpions and their venom components are effective in treating numerous illnesses, such as cardiovascular diseases and cancer [20, 21] due to their antimicrobial, anticancer, and analgesic properties [22]. Giovannini et al. (2017) state that Vidatox, *Junceus rhopalurus* venom, is presently being tested in clinical studies as a potential treatment for hepatocellular cancer [23]. In this context, current innovations in the field of proteomics contribute to the in-depth understanding of this diversity. Using techniques such as mass spectrometry, numerous venom components have been identified and characterized with high precision. For instance, proteomic analyses of scorpion venoms such as those from *Mesobuthus martensii* and *Tityus serrulatus* have revealed a wide array of peptides, including various neurotoxins, antimicrobial peptides, enzymes, and novel isoforms of known toxins, and previously unreported peptides [24]. Our proteomic analyses also proved that *Scorpio fuscus* venom (*SFV*) possesses 82 different proteins/peptides as channel inhibitors or toxins such as Maurocalcin, Opiscorpine-1, Toxin-like peptide, Phospholipase A2 enterotoxin, Phospholipase A2 imperatorin-1 [25]. All of these findings expand our knowledge of venom composition while providing insights into the evolutionary adaptations of scorpions and potential therapeutic applications of venom-derived peptides.

Building on this knowledge, it becomes essential to explore not only the compositional diversity of scorpion venoms but also their functional relevance in cancer therapy. Therefore, the present study aims to investigate the effects of *SFV* on human CRC cells and elucidate the molecular mechanisms involved in CRC signaling and apoptotic pathways in both in vitro and in vivo orthotopic colon cancer mice model. This study may contribute to developing next-generation biotherapeutic approaches by revealing the potential effects of *SFV* components in treating colorectal carcinoma.

## Methods

### Collection of scorpion specimens, obtaining and storage of scorpion venoms

*Scorpio fuscus* specimens, which were used for milking venom, were collected from Kozan District of Adana Province (Eski Mantaş Village, 37°31′30″N, 35°54′22″E, 434 m and Eski Kabasakal Village, 37°26′09″N, 35°51′23″E, 158 m) from 08 to 14 July 2021 and from 07 to 16 July 2022. Scorpions were kept in captivity in 5 L containers, including cocopeat soil and an egg container for hiding. Specimens were fed crickets, grasshoppers, and larvae of *Tenebrio molitor*. Specimens were milked after carbon dioxide anesthesia and 12 V/25A electrical stimulation was used for venom extraction. Specimens were milked once a month. The obtained venom was stored at − 80 °C.

### Characterization of venom

One mg of the lyophilized *SFV* was dissolved in 1 mL of 0.1% saline solution, the mixture was centrifuged, and the supernatant was used for further analyses. The pH, protein concentration, purity, and peptide profile of the *SFV* were determined to characterize the venom by pH meter, Bicinchoninic acid (BCA) method, UV–visible spectrophotometer, and high-performance liquid chromatography (HPLC), respectively.

#### UV–vis spectrum analyses

Spectra analysis of venom was performed using Shimadzu brand UV-1900i model spectrophotometer device. Spectrum scanning was performed between 190 and 400 nm wavelengths. A venom-specific maximum peak was observed at 280 nm.

#### Determination of protein concentration

The concentration of protein in *SFV* was determined by a colorimetric BCA assay. Samples were measured at 562 nm after half an hour of incubation at 37 °C. Bovine serum albumin (BSA) was used as a standard at concentrations of 1500, 1000, 750, 500, 250, 125, 100, 50, 25, and 0 μg/mL.

#### Chromatographic profiles

One milligram of *SFV* was dissolved in 500 µL of solvent A (0.1% trifluoroacetic acid (TFA) in ultrapure water), passed through a 0.45-µm sterile filter (Millex®-HP Syringe Filter Unit, polyethersulfone, 33 mm, gamma-sterilized), and transferred into 1.5 mL amber HPLC vials. Chromatographic analyses were performed using a Shimadzu Prominence-i LC-2030 3D HPLC system. Separation of *SFV* components was achieved using a reverse-phase C18 column (250 mm × 4.6 mm inner diameter, 5 µm particle size), maintained at a constant column temperature of 30 °C. The flow rate was set to 1.0 mL/min, and the total run time was 60 min. A linear gradient elution was applied using solvent A (0.12% TFA in water) and solvent B (0.10% TFA in acetonitrile), starting from 40% solvent A and 60% solvent B. Eluted compounds were detected using a photodiode array (PDA) detector, and chromatograms were recorded at λ = 280 nm for quantitative analysis.

#### Electrophoretic profiles

Electrophoretic profiles of the venom were determined using Sodium Dodecyl Sulfate–Polyacrylamide Gel Electrophoresis (SDS-PAGE) in a Mini-PROTEAN-Bio-Rad electrophoresis system. One micrograms/microliter of the venom was loaded and separated under standard conditions of 10 mA for the stacking gel (4%) and 20 mA for the separating gels (10%) for 3 h in ERB (Tris–EDTA, borate, and NaCl). The protein bands were stained with Coomassie brilliant blue R-250. The visualized protein bands were imaged using an imaging system (G:BOX Chemi XRQ, SynGene). Molecular weights were estimated using standard high-range markers (10–250 kDa) (Bio-Rad).

### In vitro studies

#### Cell lines, culture conditions, and cytotoxicity analyses

Human CRC cell lines (DLD-1, HT-29, Caco-2) and human healthy colon epithelial cell line (CCD-18Co) were purchased from the American Type Culture Collection (ATCC, Rockville, MD). The cells were cultured in RPMI-1640, McCoy’s 5 A, and EMEM, respectively, supplemented with 10% FBS and 2 mM L-glutamine in a 37 °C and 5% CO_2_ incubator. Cells were seeded at a density of 1 × 10^4^ cells per well in 96-well plates and allowed to attach for 24 h before treatment. After cell attachment, cells were treated with varying concentrations of *SFV* (0–250 µg/mL) for 48 h. Following treatment, the cells were washed with phosphate-buffered saline (PBS), and cell viability was measured using Alamar Blue reagent (Invitrogen, Thermo Fisher Scientific, Waltham, MA, USA) after a 2-h incubation. IC_50_ values were calculated from sigmoidal dose–response curves.

#### Wound healing assay

In vitro cell migratory capacity was evaluated using the wound healing assay. Briefly, CRC cells were seeded into 6-well plates and allowed to reach approximately 90% confluence. A uniform scratch was created in the cell monolayer using a sterile 200 µL pipette tip. After washing with PBS to remove detached cells, fresh culture medium containing *SFV* at the IC₅₀ concentration was added. Wound closure was monitored and imaged at 0, 12, 24, and 48 h using an inverted phase-contrast microscope. The wound area was quantified using ImageJ software, and migration rates were calculated as the percentage of wound closure relative to the initial wound width at 0 h.

#### Colony formation assay

To detect the effects of the *SFV* on cells’ colony formation potency, a soft agar colony formation assay was conducted. A single-cell suspension (3 × 10^4^ cells/well) was seeded into 24-well plates and incubated with an equivalent concentration of IC_50_ value, and dead cells were removed after 48 h. Base Agar (1%) was mixed with growth medium and loaded into 6-well plates as the base layer. Then, 0.7% top agar was mixed with the cell suspension, and the mixture was added on top of the base agar. Finally, the cell growth medium was added and incubated at 37 °C and 5% CO_2_ for 15 days. After incubation, cells were stained with 0.01% (w/v) crystal violet, and colony numbers were calculated with Image J software.

#### Apoptosis assays

Apoptotic rates of CRC cells were evaluated using the PE Annexin V Apoptosis Detection Kit (BD Biosciences, USA). Cells were treated with *SFV* at IC₅₀ concentrations for 48 h. After treatment, cells were harvested, stained with Annexin V-APC and 7-AAD for 20 min in the dark, and analyzed using a BD flow cytometer. At least 10,000 events per sample were recorded, with doublets and debris excluded via forward/side scatter gating. Apoptotic populations were quantified as early (Annexin V⁺/7-AAD⁻) and late (Annexin V⁺/7-AAD⁺) apoptosis. Experiments included three independent biological replicates, each performed with two technical replicates.

#### mRNA expression analysis in apoptotic and colon cancer progression pathways

qRT-PCR was performed to assess gene expression changes in apoptotic and CRC pathways. Cells were treated with the *SFV* at its IC_50_ concentration and incubated for 24 h. Total RNA was extracted using TRIzol and quantified via NanoDrop™ 2000. cDNA was synthesized with the iScript cDNA Synthesis Kit. Expression changes were analyzed using Human Colorectal Cancer and Apoptosis Panels. The 2^−ΔΔCt^ method was used for relative quantification, with GAPDH, GUSB, PPIA, B2M, HPRT1, PGK1, ACTB, and RPL13A serving as internal control genes. Primer specificity was verified using Primer3 and NCBI BLAST.

#### Protein expression analysis in apoptotic and colon cancer progression pathways

Western blot analysis was performed to evaluate the effects of *SFV* on CRC signaling pathways and apoptosis-related protein expression. Cells were treated with *SFV* at the IC₅₀ concentration and incubated for 48 h. Total protein extracts were separated by SDS–PAGE (7.5–12%) and transferred onto polyvinylidene difluoride (PVDF) membranes. Membranes were blocked with 5% non-fat dry milk and incubated overnight at 4 °C with the following primary antibodies: Bax (50,599–2-Ig, 1:8000), Bcl-2 (26,593–1-AP, 1:2,000), Caspase-3 (25,128–1-AP, 1:1000), p53 (10,442–1-AP, 1:3000), BRAF (20,899–1-AP, 1:2000), MLH-1 (11,697–1-AP, 1:2000), and NF-κB (33,259–1-AP, 1:1000). GAPDH (10,494–1-AP, 1:10,000) was used as the internal reference protein due to its stable expression under the experimental conditions. After washing, membranes were incubated with HRP-conjugated secondary antibodies (Goat Anti-Rabbit IgG, SA00001-2, 1:10,000; Goat Anti-Mouse IgG, SA00001-1, 1:10,000) for 2 h at room temperature. Protein bands were visualized using an enhanced chemiluminescence (ECL) detection system (Syngene G:Box imaging system). Densitometric analysis was performed using GeneSys imaging software. The exposure time for band detection ranged from 30 s to 3 min, depending on the signal intensity of the target proteins.

### In vivo* studies*

All experimental procedures were approved by the Selcuk University Animal Experiments Local Ethics Committee (Protocol No. 2019–42) and conducted in strict accordance with the European Directive 2010/63/EU on the protection of animals used for scientific purposes, the Guide for the Care and Use of Laboratory Animals, and the ARRIVE 2.0 guidelines [26]. All animals were obtained from and housed at the Selcuk University Experimental Medicine Research and Application Center (SÜDAM, Konya, Turkey).

#### Dose-range studies

Acute toxicity of *SFV* was assessed using a graded dose–response design to determine lethality and guide subsequent xenograft studies. Twelve-week-old male and female Swiss albino mice (*n* = 10 per group; 5 per sex) were acclimated for 7 days under standard housing conditions with ad libitum access to food and water.

Initial dose selection was guided by extrapolation from in vitro cytotoxicity data using the regression model described by Spielmann et al. (1999) and subsequently adopted in ICCVAM evaluations [27, 28], according to the following equation: log₁₀(LD₅₀, mg/kg) = 0.372 × log₁₀(IC₅₀, μg/mL) + 2.024. The experimentally determined IC₅₀ value of 14.8 μg/mL yielded log₁₀(LD₅₀) = 2.459, corresponding to a theoretical oral LD₅₀ of approximately 288 mg/kg. This estimate was used to inform starting-dose selection, with final dose levels refined through preliminary range-finding studies and route-specific considerations for intraperitoneal (IP) administration. Animals were weighed prior to dosing (mean 20 g, range 18–22 g). Initial dose selection was informed by OECD Test Guideline 425, in vitro cytotoxicity data (IC₅₀ = 14.8 μg/mL), and the regression model of Spielmann et al. (1999), yielding a theoretical oral LD₅₀ of ~ 288 mg/kg.

Lyophilized venom was dissolved in sterile saline (16 mg/mL) and serially diluted to deliver 0.1–1.6 mg/mouse in 0.1 mL via IP injection. Animals were randomly assigned to treatment groups using a computer-generalized randomization sequence (Graphpad Prism 10.0). Control animals received saline. Injections were administered in conscious mice using a 26-gauge needle during the light phase (09:00–12:00). Animals were monitored continuously for 1-h post-injection, at 30-min intervals for 6 h, and every 12 h up to 48 h. Clinical signs recorded included respiration, posture, locomotion, tremor, ocular changes, social behavior, and food/water intake. Humane endpoints were predefined according to international guidelines. Surviving animals were euthanized under ketamine/xylazine anesthesia followed by cervical dislocation and necropsy. Observed mortality across dose groups yielded a median lethal dose (LD₅₀) of 0.496 mg/mouse, which was used to define sublethal doses for subsequent xenograft experiments.

#### Xenograft models

To mimic human CRC, NOD/SCID mice were selected as orthotopic xenograft models. Male NOD/SCID mice (*n* = 30; 6–8 weeks old, 20–25 g) were housed at the Selçuk University Experimental Medicine Research and Application Center (SUDAM) in a climate-controlled room (55% ± 5% relative humidity, 22 °C ± 2 °C, and 12/12-h light/dark cycle) in individually ventilated cages (IVC) of the NexGen Thermo IVC cage systems (Allentown, NJ, USA). Mice were housed individually and provided with sterile drinking water and pellet feed (Standard Diet 1320; Altromin, Lage, Germany) ad libitum. All procedures performed outside the cages were conducted under a laminar flow hood (CS5 changing station; Techniplast, Buguggiate, Italy).

#### In vivo tumor implantation and treatment 

Animals were randomly assigned into three groups: Control (sham-operated; *n* = 10), Colon cancer (CDX; *n* = 10), and CDX + *SFV* (CDX + *SFV*; *n* = 10). In the CDX and CDX + *SFV* groups, an orthotopic colon xenograft (*n* = 4) was established by subepithelial injection of a suspension containing 4 × 10^6^ DLD-1 human colorectal adenocarcinoma cells (ATCC® CCL-221™) into the distal colon, while a subcutaneous xenograft (*n* = 6) was placed under anesthesia in the right subscapular region. During weeks 3 and 5 post-engraftment of DLD-1 cells, intraperitoneal administration of 16.53 mg/kg *SFV* was performed for the orthotopic subgroups, and both IP and intratumoral routes were used for the subcutaneous subgroups. Control animals received vehicle administration under identical experimental conditions, including injection schedule and handling procedures, and served as procedural (sham) controls.

#### Quantitative analysis of tumor growth

Tumor development was monitored twice a week by manual examination, palpation, and Vernier caliper measurements. Tumor volumes at weeks 2, 4, and 6 were calculated using the standard ellipsoid formula: V = π/6 × (length × width × height). At the end of the experiment, mice were euthanized under deep anesthesia, and tumors were excised, weighed, and photographed for quantitative comparison among groups.

#### Histopathological examination

During necropsy, all tumors and adjacent normal tissues were collected. Samples were fixed in 10% neutral buffered formalin for 48 h, embedded in paraffin, and sectioned at 4 µm thickness. Sections were stained with hematoxylin and eosin (H&E) for evaluation of tumor architecture, necrosis, and cellular morphology under light microscopy. Histomorphometric analyses were performed to assess necrotic areas and viable tumor cell density. Immunohistochemical assays were subsequently applied to detect apoptotic and oncogenic markers (Bax, Bcl-2, p53, KRAS, NF-κB).

### Statistical analyses

All experiments were performed in triplicate, and all analyses were conducted using SPSS software version 23.0 (IBM Corp., Armonk, NY, USA). Normality was evaluated using the Kolmogorov–Smirnov test. For comparisons between two groups, independent *t*-tests were applied, while differences among more than two groups were assessed via one-way ANOVA followed by Tukey’s post hoc analysis. Data are presented as mean ± standard deviation (SD). Results with *p*-values < 0.05 were considered statistically significant. IC₅₀ values are reported together with their 95% confidence intervals.

## Results

**Fig. 1:**
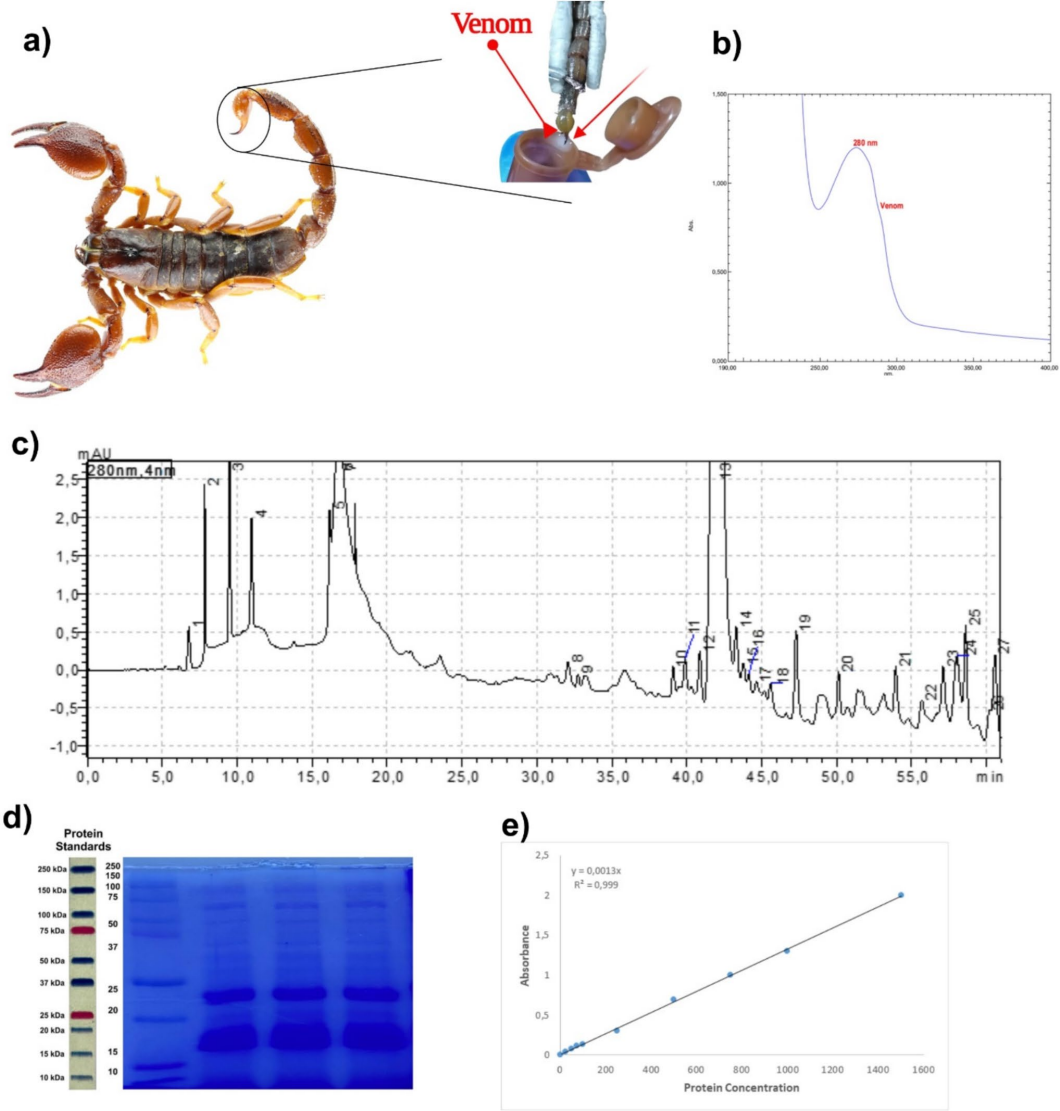
Collection of *Scorpio fuscus* specimens and biochemical characterization of scorpion venom. **a** Venom extraction process from *Scorpio fuscus* specimens using 12 V/25A electrical stimulation under CO₂ anesthesia. **b** UV–vis spectrum analysis of venom extracted from *Scorpio fuscus* specimen. A venom-specific maximum peak was observed at 280 nm. **c** High-performance liquid chromatography (HPLC) chromatogram of venom from *Scorpio fuscus* specimen. The eluents were delivered to a PDA detector and quantitative analysis was performed at a wavelength of λ = 280 nm. **d** Electrophoretic profile of venom from *Scorpio fuscus* specimens obtained by ERB buffer system. The protein bands were stained with Coomassie brilliant blue R-250 and visualized using an imaging system (G:BOX Chemi XRQ, SynGene). Molecular weights were estimated using standard high range (10–250 kDa markers standards) (Bio-Rad). **e** Determination of protein concentration by Bicinchoninic Acid (BCA) method. Bovine serum albumin (BSA) was used as a standard at different concentrations (μg/mL). Absorbance = 562 nm. *R*.^2^ = 0.999

Venom was successfully extracted from *S. fuscus* specimens using electrical stimulation under CO₂ anesthesia (Fig. 1a). UV–Vis spectrophotometric analysis revealed that the venom exhibited a characteristic absorption peak at 280 nm (protein absorption), indicating the presence of protein-rich constituents (Fig. 1b). The prominent and clearly detectable bands appeared at 12–15 kDa, 20–25 kDa, 40–45 kDa, and within the high-molecular-weight range of 70–100 kDa. HPLC analysis displayed a distinct chromatographic profile with multiple elution peaks, reflecting the biochemical complexity of the venom (Fig. 1c). SDS–PAGE demonstrated a wide distribution of protein bands ranging from ~ 10 to 250 kDa, confirming the heterogeneity of venom proteins (Fig. 1d). Protein quantification by BCA assay determined a concentration of 1.2 ± 0.1 mg/mL (Fig. 1e).

**Fig. 2:**
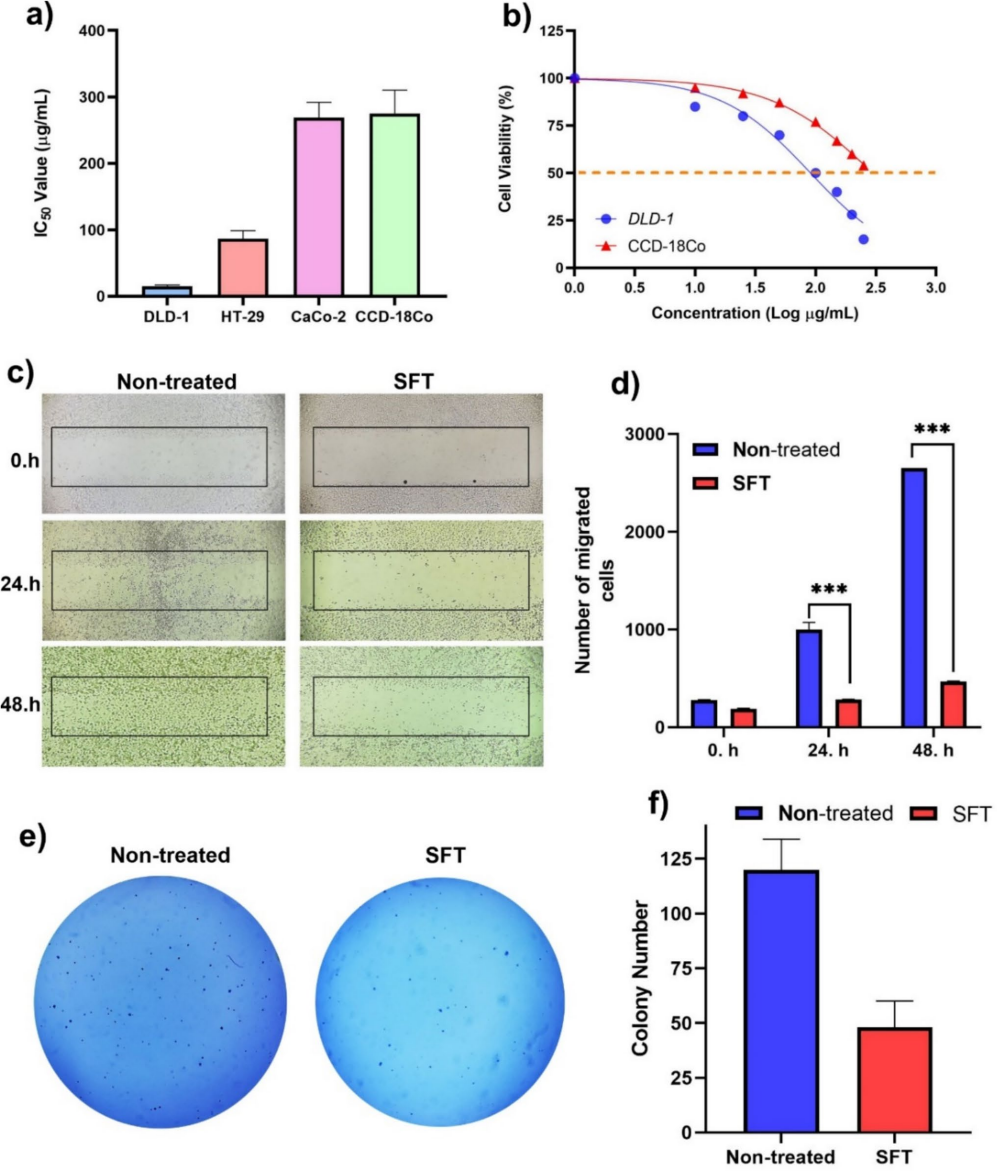
Cytotoxic and anti-proliferative effects of venom from *Scorpio fuscus* specimens on colorectal carcinoma and healthy cells. **a** IC₅₀ values (µg/mL) of venom from *Scorpio fuscus* specimens obtained by Alamar Blue cytotoxicity assay on colorectal cancer DLD-1, HT-29, and CaCo-2 cell lines, and normal colon fibroblast CCD-18Co cell line. **b** Concentration-dependent percentage of cell viability on colorectal cancer DLD-1 and normal colon fibroblast CCD-18Co cells treated with venom from *Scorpio fuscus* specimen. **c** Representative images of the wound healing assay at 0 h, 24 h, and 48 h for non-treated cells, and cells treated with *Scorpio fuscus* venom (SFT). **d** The number of migrated cells at 0 h, 24 h, and 48 h for non-treated cells, and cells treated with *Scorpio fuscus* venom (SFT). Data are presented as mean ± SD (*n* = 3). ***p < 0.001, compared to the non-treated group. **e** Colony formation assay of *Scorpio fuscus* venom on non-treated and cells treated with *Scorpio fuscus* venom (SFT). (f) Number of colonies on non-treated and cells treated with *Scorpio fuscus* venom (SFT) cells. Data are presented as mean ± SD (*n* = 3)

**Fig. 3:**
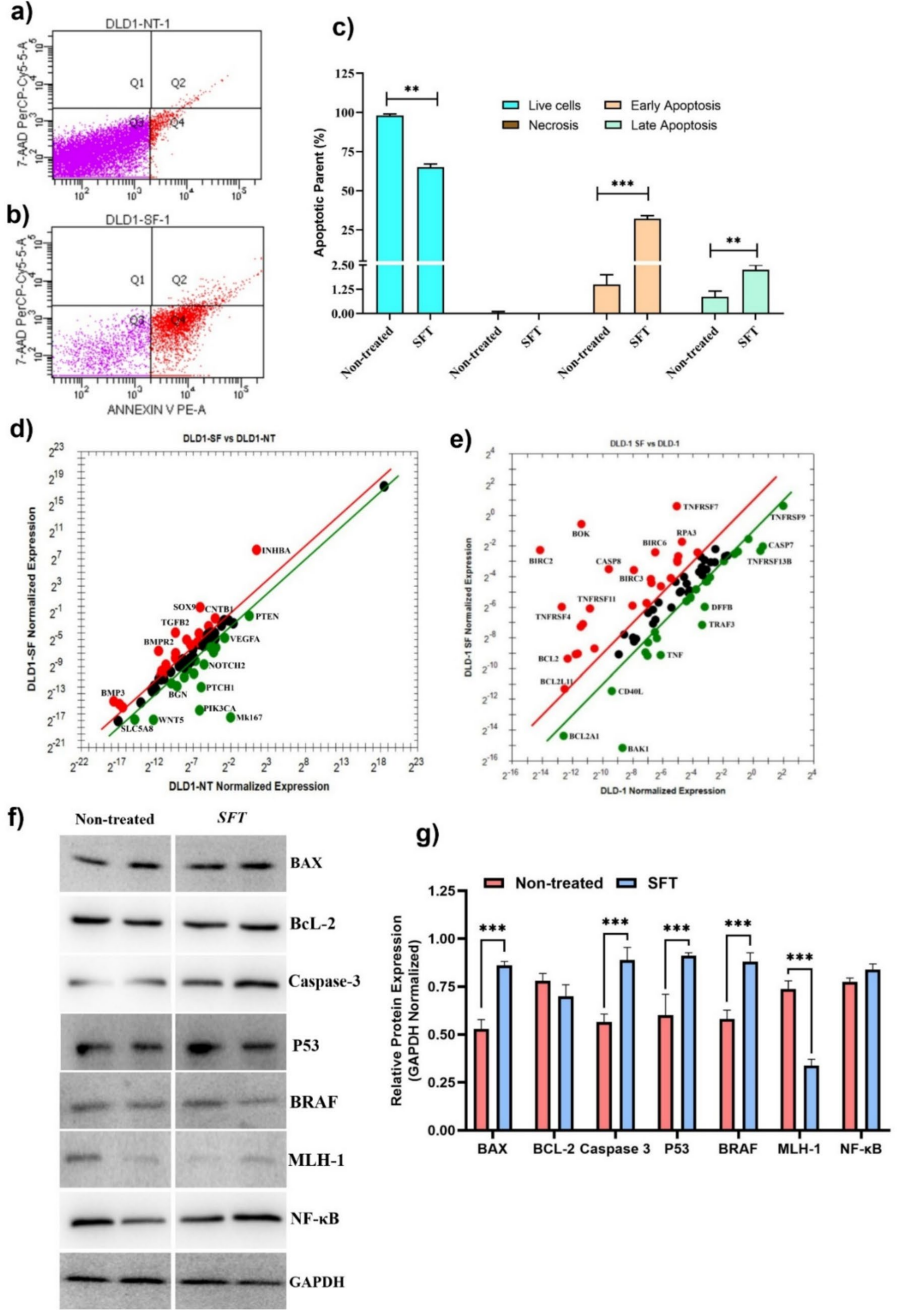
Effects of SF venom on mRNA and protein expressions of colorectal carcinoma signaling and apoptotic pathways. Flow cytometric analysis on **a** non-treated and **b** *Scorpio fuscus* venom treated (SFT) cells. **c** Comparison of live cells, necrosis, early apoptosis, and late apoptosis rates (%) for non-treated and cells treated with *Scorpio fuscus* venom (SFT). Data are displayed as the mean ± standard deviation (SD). **p < 0.01, ***p < 0.001, compared to the non-treated group. **d**, **e** Gene expression analysis of colorectal cancer and apoptosis-related genes on non-treated (DLD1-NT) and *Scorpio fuscus* venom-treated (DLD1-SF) cells using colorectal cancer and apoptosis panels. **f** Representative protein bands (**g**) analyses of protein expressions following SFT. Q1: cells undergoing necrosis (Annexin −, 7-AAD +); Q2: cells in late stages of apoptosis (Annexin +, 7-ADD +); Q3: Living cells (Annexin −, 7-ADD −); Q4: cells in early stages of apoptosis (Annexin +, 7-ADD −)

The cytotoxic potential of *SFV* was assessed in CRC (DLD-1, HT-29, and CaCo-2) and normal colon fibroblast CCD-18Co cells. The venom induced a dose-dependent reduction in cancer cell viability, with IC₅₀ values of 14.8 µg/mL for DLD-1, 26.4 µg/mL for HT-29, and 32.7 µg/mL for CaCo-2 cells. In contrast, the IC₅₀ for CCD-18Co cells exceeded 250 µg/mL, indicating selective cytotoxicity toward cancerous cells (Fig. 2a, b). Functional assays confirmed the anti-metastatic potential of the *SFV*. Wound-healing assays demonstrated an 84% reduction in cell migration at 48 h compared with untreated controls (Fig. 2c, d). In addition, colony formation assays revealed a significant 49% decrease in clonogenic survival of venom-treated cells relative to untreated cells (Fig. 2e, f). *SFV* treatment was associated with a significant increase in apoptotic cell populations, as determined by flow cytometric analysis. Compared with untreated controls, venom-treated DLD-1 cells displayed a marked increase in early (Annexin V⁺/7-AAD⁻) and late (Annexin V⁺/7-AAD⁺) apoptosis, alongside reduced viable cell percentages (Fig. 3a–c). These findings indicate that *SFV* induces apoptotic responses at a phenotypic level in CRC cells. Gene expression analysis further revealed modulation of multiple apoptosis-related genes following *SFV* exposure, supporting the involvement of apoptotic signaling processes without implying direct activation of specific apoptotic pathways. Differential gene expression analysis identified a total of 51 genes exhibiting at least a two-fold change compared to the control group. Among these genes, seven were significantly upregulated (BIRC2, BIRC3, BIRC6, CASP8, TNFRSF8, TNFRSF11, and BOK) and two were significantly downregulated (BAK1 and TRAF3) based on an adjusted *p* value < 0.05 (Fig. 3d, e). The remaining genes exhibiting ≥ twofold changes did not reach statistical significance after multiple testing correction. Western blot analysis confirmed protein-level modulation of apoptosis and CRC signaling markers, consistent with transcriptional changes (Fig. 3f, g).

**Fig. 4:**
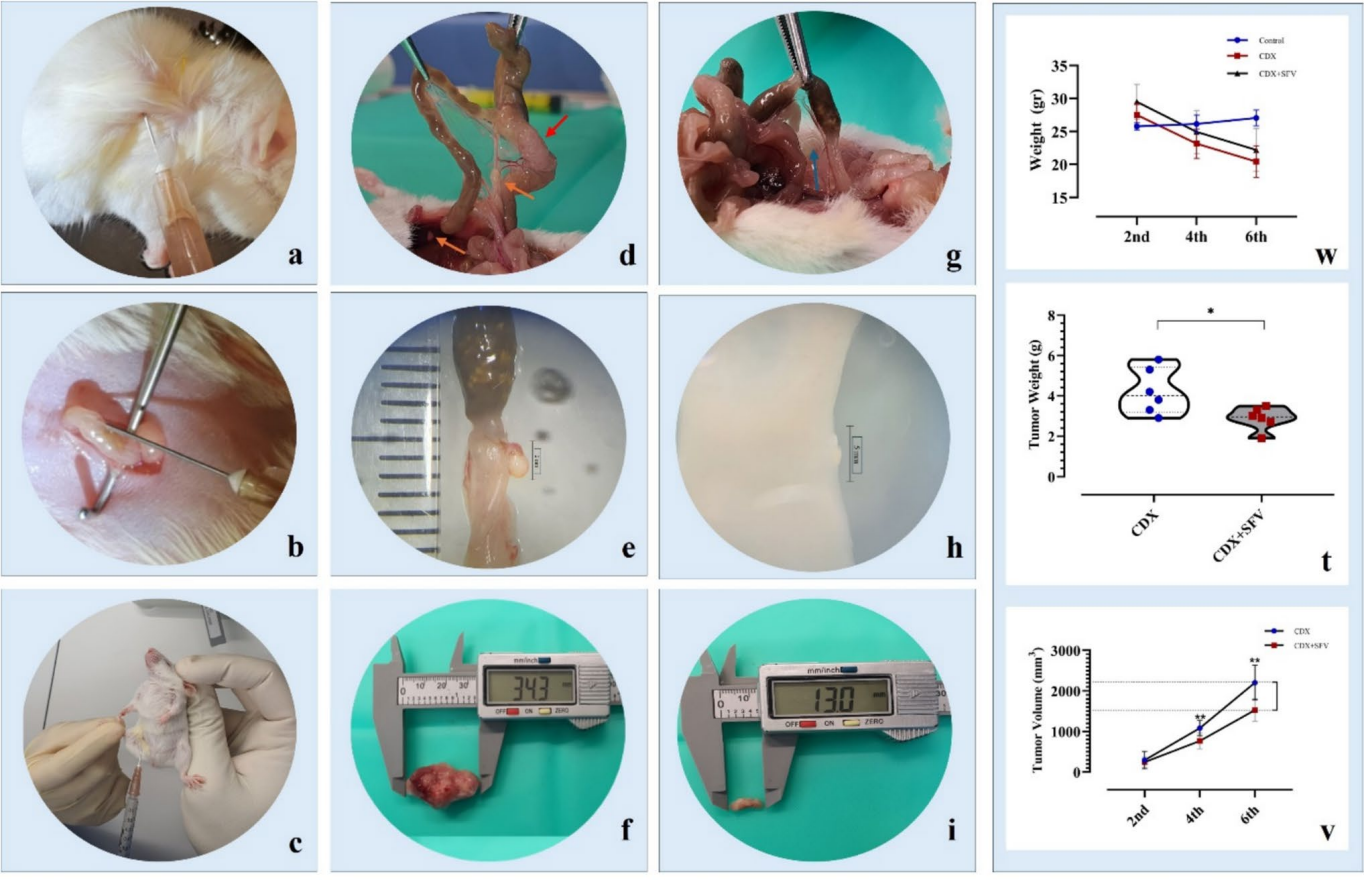
In vivo experimental procedures (**a**–**c**) and necropsy findings (**d**–**i**), with statistical analysis (**w**–**v**) in groups (**d**–**e** CDX; **g**–**i** CDX + *SFV*). **a** Subcutaneous implantation of DLD-1 cells into the right subscapular region. **b** Orthotopic implantation into the subepithelium of the distal colon. **c** Treatment procedures at the 3rd and 5th weeks via intraperitoneal injection of *Scorpio fuscus* venom (*SFV*). **d**–**e** CDX group necropsy. **d** The red arrow indicates the primary tumor focus, while the orange arrows indicate metastatic foci (top: mesenteric lymph node; bottom: liver). **e** Microscopic examination revealed that the primary tumor had reached 2 mm in length, was firmly attached to the subepithelium, and exhibited distinguishable blood vessels. **g**–**i** CDX + *SFV* group necropsy. **g** On macroscopic inspection, the primary neoplasm was regressed and faintly delineated; no macroscopic metastatic foci were observed, and the mesentery displayed an opalescent, ground-glass appearance. **h** Microscopic evaluation showed that the primary tumor had regressed to less than ~ 0.5 mm. **f** and **i** Tumor dimensions were significantly reduced following *SFV* treatment

Following subcutaneous inoculation of DLD-1 cells into the right scapular region (Fig. 4a), all mice developed progressively enlarging tumors in the CDX group, with mean tumor volumes reaching 2205.39 ± 427.78 mm^3^ by week 6. Tumor development was first detected 7–10 days post-implantation and monitored by inspection, palpation, and caliper-based measurements. Volumes were calculated using the 3D ellipsoid formula (V = π/6 × L × W × H) [29]. The detailed results are shown in Table 1. Tumor volumes at week 2 did not significantly differ between groups. However, differences in growth rates were noticeable by week 4. Both CDX and CDX + *SFV* groups displayed significant intragroup increases in tumor size (*p* < 0.01), yet treatment with *SFV* markedly attenuated progression. At week 6, mean tumor volume in the CDX + *SFV* group (1528.55 ± 277.39 mm^3^) was reduced by 30.69% relative to the CDX alone (*p* = 0.008), indicating a biologically relevant inhibitory effect (Fig. 4v).

**Table 1:** Tumor volume analyses (mm^3^)

Group	CDX (mean ± SD)	CDX + *SFV* (mean ± SD)
2nd week	293.44 ± 214.85 (a)	243.43 ± 122.33 (a)
4th week	1083.82 ± 186.57 (b)	766.98 ± 202.36 (b)
6th week	2205.39 ± 427.78 (c)	1528.55 ± 277.39 (c)

^*^Lowercase letters (a, b, c) indicate intragroup differences in tumor weights (*n* = 6)

At necropsy, subcutaneous tumors were resected with intact margins and weighed (Fig. 4f, i). The mean tumor weight in the CDX + *SFV* group (2.88 ± 0.56 g) was significantly lower than that in the CDX group (4.22 ± 1.13 g), corresponding to a 68.25% reduction (*p* < 0.05) (Table 2). Remarkably, necroscopic findings revealed widespread metastatic foci in the lymph nodes, mesentery, liver, kidneys, diaphragm, and lungs of CDX mice (Fig. 4d, e), whereas only mild mesenteric ground-glass opacification was detected in CDX + *SFV* animals (Fig. 4g, h).

**Table 2:** Absolute tumor weights (g)

Group	CDX (mean ± SD)	CDX + *SFV* (mean ± SD)
Tumor weight (g)	4.22 ± 1.13 (a)	2.88 ± 0.56 (b)

^*^Lowercase letters (a, b, c) indicate intragroup differences in tumor weights (*n* = 6)

**Fig. 5:**
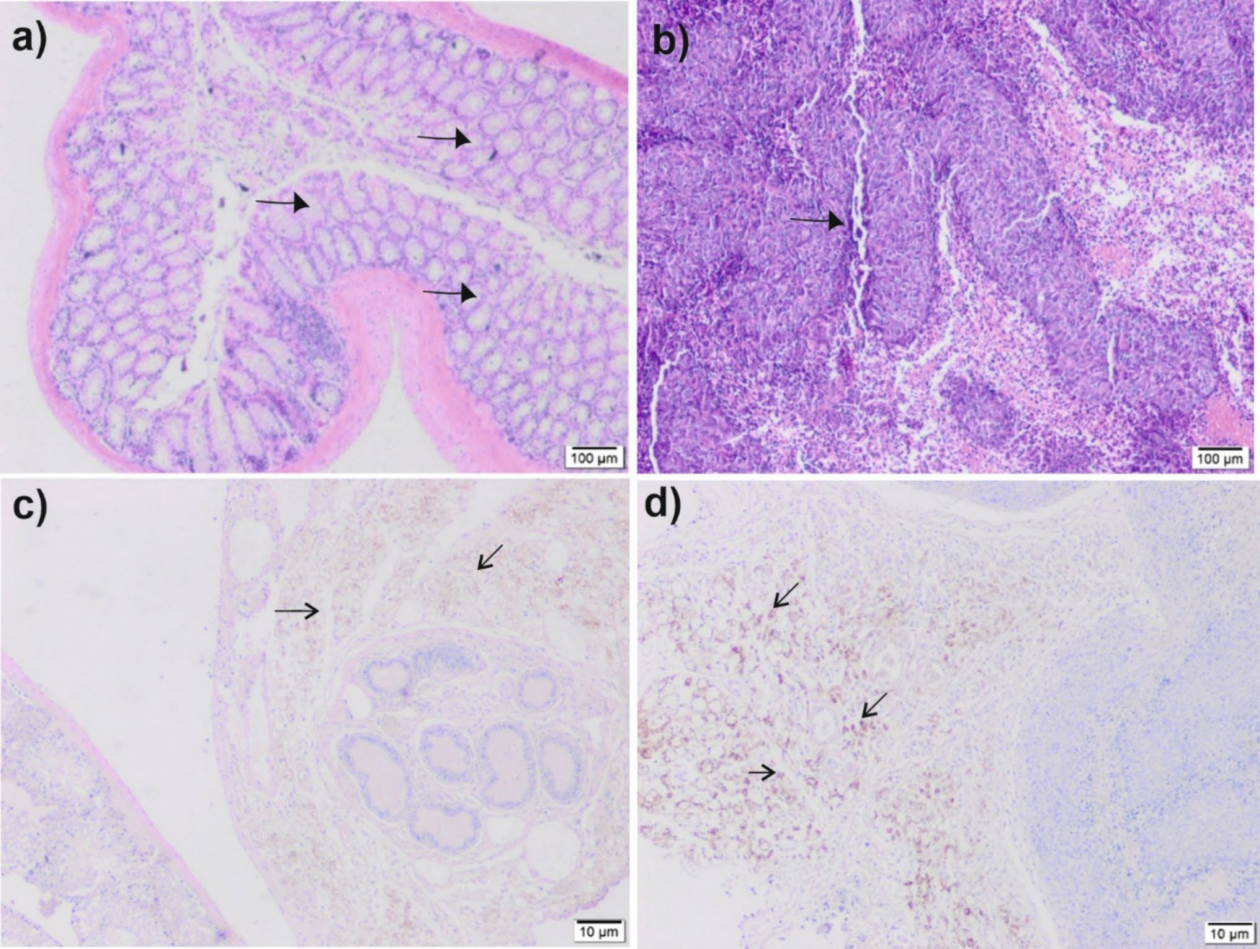
Histopathology and immunohistochemistry (IHC) analyses. **a** Representative H&E-stained section from the control group showing normal intestinal architecture with preserved crypt structures (arrows). **b** H&E-stained section from the CDX group displaying disrupted tissue architecture, densely packed malignant epithelial cells, and invasive tumor margins (arrow). **c** Immunohistochemical staining for Bax in tumor tissues from the CDX + *SFV* group, demonstrating increased pro-apoptotic protein expression (arrows). **d** Immunohistochemical staining for BcL-2 in tumor tissues from the CDX group, indicating strong anti-apoptotic protein expression (arrows). Scale bars: 100 µm (**a**, **b**), 10 µm (**c**, **d**)

Histological examination supported the volumetric and weight findings. Tumors in control mice were characterized by densely packed malignant cells, frequent mitoses, and invasive borders. In contrast, CDX + *SFV* tumors exhibited extensive necrotic areas, diminished nuclear pleomorphism, and reduced mitotic activity. Immunohistochemical staining was performed to evaluate apoptotic and oncogenic markers in xenograft tumor tissues. In control tumors, BcL-2 expression was strong and diffuse, whereas Bax and p53 staining were weak or absent. In contrast, *SFV* treatment induced a dose-dependent shift toward a pro-apoptotic profile (Fig. 5a–d). Both the medium-dose (10 mg/kg) and high-dose (20 mg/kg) groups showed markedly increased Bax and p53 expression, accompanied by reduced Bcl-2 levels. Quantitative immunostaining scores revealed statistically significant differences between control and *SFV*-treated groups for both KRAS and NF-κB (*p* < 0.05). These findings indicate a biologically relevant alteration in marker expression associated with *SFV* treatment. However, as immunohistochemistry provides semi-quantitative information, these changes should be interpreted as evidence of modulated protein expression rather than definitive proof of functional inhibition of the corresponding signaling pathways.

## Discussion

The global rise in colorectal cancer underscores the urgent need for novel and alternative therapeutic approaches. Epidemiological projections estimate that worldwide colorectal cancer-related mortality will increase from approximately 538,000 cases in 2022 to nearly 1 million by 2045 [30]. In this context, bioactive peptides and proteins derived from scorpion venom have been suggested to selectively modulate key oncogenic pathways, offering a promising avenue for targeted therapeutic development.

*Scorpio fuscus*, a member of the Scorpionidae family, possesses a remarkably rich venom composition [25, 31].

Particularly low molecular weight peptides and proteins in the venom can directly influence its biological activity [32]. Analytical methods, such as spectrophotometric profiling, are frequently applied to characterize venom composition. The milking technique affects the UV-absorption spectra. Venoms obtained via electrical stimulation show a single peak at 280 nm, indicating a purer, protein-rich composition, whereas manually collected venoms usually show three peaks (280 nm, 220–380 nm, and 520–600 nm), reflecting the presence of both protein and non-protein components such as biogenic amines, nucleotides, pigments, or oxidized metabolites released from glandular cells [33, 34]. *SFV* has a wide distribution of protein bands ranging from ~ 10 to 250 kDa [25]. The electrophoretic profile of venom demonstrated that the 12–15 kDa bands likely correspond to low-molecular-weight peptides, including neurotoxic and cytolytic components commonly reported in scorpion venoms [35]. The 20–25 kDa region contained abundant proteins, potentially representing ion channel-modulating toxins and enzymes [36]. Intermediate bands (~ 30–45 kDa) may include proteolytic enzymes [37], while high-molecular-weight fractions (> 70 kDa) reflected larger enzymatic proteins and structural components [38]. The presence of both low- and high-molecular-weight fractions confirmed the biochemical heterogeneity of *SFV*. Notably, the intensity of the low-molecular-weight bands suggested enrichment of bioactive peptides, which could underlie the selective cytotoxic effects observed in colorectal carcinoma cells. In our previous study, we demonstrated that *S. fuscus* possesses 88 different peptides/proteins with a rich array of bioactive compounds, ranging from potassium channel blockers (like maurotoxin and α-KTx6.2) to calcium-binding peptides and phospholipase A₂ isoforms [25]. Densitometric analysis in the present study further demonstrated that proteins in the 12–25 kDa range represented the most abundant fraction of the venom proteome, highlighting their likely contribution to biological activity (Fig. 1d).

Previously identified molecules are highly relevant to cancer biology. For example, Kv channel inhibitors are known to slow tumor growth and needle metastatic potential, as dysregulated expression of channels such as Kv1.3, Kv10.1, and hERG (Kv11.1) is frequently observed in colorectal cancer [39, 40]. Another important group of venom molecules are phospholipase A₂ enzymes. Similar proteins from other scorpions, such as hemilipin from *Hemiscorpius lepturus* and PLA₂s from *Scorpio maurus*, have been shown to inhibit angiogenesis by interfering with integrin signaling [41, 42]. These processes may underlie the anticancer effects observed with scorpion venoms, including those reported in our study.

Our in vitro results demonstrated that *SFV* possesses selective and multifaceted antitumor activity against CRC cells. The venom caused a dose-dependent decrease in cell viability in DLD-1, HT-29, and CaCo-2 cell lines, while exhibiting minimal effects on normal CCD-18Co fibroblasts with IC₅₀ values exceeding 250 µg/mL. This observed difference in cytotoxicity highlights the therapeutic selectivity of scorpion venom towards malignant cells and is consistent with previous studies [43–46].

In addition, *SFV* reduced colony formation by nearly half and suppressed migration capacity by 84% compared to untreated controls. This result indicates that venom components may not only inhibit primary tumor growth but also reduce the metastatic potential of CRC cells by disrupting two critical features of malignant progression: colony formation and migration. Significant increases in both early and late apoptotic cell populations in CRC cell lines (DLD-1, HT-29, and CaCo-2) obtained from flow cytometry suggest that the *SFV* also triggers the activation of programmed cell death pathways. Similar results have been obtained in the literature with venoms from different scorpion species. For example, Canak et al. (2025) demonstrated that *Mesobuthus eupeus* venom significantly inhibited cell migration and colony formation in DLD-1 and HT-29 CRC cells, affected the modulation of metastasis-related signaling pathways, and induced apoptosis [47].

*SFV* led to the upregulation of both CASP8 and BOK genes, which are important pro-apoptotic regulators, compared to untreated controls. CASP8, a key initiator of extrinsic apoptosis, has been reported as a promising therapeutic strategy in CRC due to its reactivation, especially considering its frequent downregulation in chemotherapy-resistant tumors [48]. BOK, a pro-apoptotic member of the BCL-2 family, contributes to mitochondrial apoptosis by promoting cytochrome c release [49]. In our study, the simultaneous downregulation of BAK1 and TRAF3 genes indicates a complex interaction between pro- and anti-apoptotic signaling, emphasizing that venom exposure does not equally alter all apoptotic regulators. Notably, the upregulation of multiple inhibitors of apoptosis (IAP) family members, including BIRC2, BIRC3, and BIRC6, suggests the presence of a potential adaptive or compensatory survival response to *SFV*-induced cellular stress. Such transcriptional responses have been previously reported in cancer cells exposed to cytotoxic agents and may reflect a dynamic balance between pro-apoptotic and pro-survival signaling mechanisms.

The upregulation of Baculoviral IAP Repeat–containing proteins (BIRC2, BIRC3, and BIRC6) in CRC cells following *SFV* exposure is consistent with an adaptive cellular response rather than definitive activation of a specific survival pathway. Members of the BIRC family are well-established inhibitors of apoptosis and are frequently implicated in apoptosis evasion and stress-induced survival signaling in cancer cells [20]. Their induction may therefore reflect a compensatory mechanism aimed at counteracting venom-induced cytotoxic stress, consistent with current models of non-genetic adaptive drug resistance, in which tumor cells transiently reprogram survival pathways to tolerate therapeutic pressure [50]. Importantly, such compensatory upregulation can coexist with pro-apoptotic signaling during early treatment responses, highlighting the dynamic balance between cell death and survival programs. While this interpretation remains hypothesis-driven in the absence of pathway-specific functional validation, it suggests that BIRC-mediated survival signaling may limit the overall therapeutic efficacy of venom exposure. Future studies should therefore investigate the functional contribution of BIRC proteins to venom-induced stress responses and explore combination strategies targeting IAP-mediated survival mechanisms to enhance apoptotic sensitivity in CRC cells.

Furthermore, the molecular changes observed in the present study provide further support for apoptosis as a key mechanism. The upregulation of CASP8 and TNFRSF family members, together with the downregulation of BAK1 and TRAF3, points to the engagement of extrinsic apoptotic pathways. Similar gene expression patterns have been reported with other scorpion venoms in colorectal carcinoma models [46, 47], reinforcing the idea that these venoms can trigger cell death through coordinated modulation of pro- and anti-apoptotic regulators. The present findings demonstrate that *SFV* induces apoptotic responses in colorectal cancer cells, as evidenced by flow cytometry and transcriptional modulation of apoptosis-associated genes. However, while changes in the expression of both pro-apoptotic and anti-apoptotic genes were observed, these results should be interpreted as phenotypic indicators of apoptosis rather than definitive activation of intrinsic or extrinsic apoptotic pathways. The results are consistent with the findings of Moradi et al. (2019), who reported that *Hemiscorpius lepturus* venom induces apoptosis in CT26 colon carcinoma cells through mitochondrial pathways and caspase activation [51]. Similarly, Al-Asmari et al. (2018a, 2018b) demonstrated that venom extracts from *Androctonus crassicauda* and *Leiurus quinquestriatus* induce apoptosis in CRC cells through the upregulation of p53, downregulation of anti-apoptotic BcL-XL, and activation of caspases [52, 53]. All these findings highlight the idea that scorpion venom peptides can target multiple apoptotic pathways, thereby enhancing their therapeutic potential. Besides, they demonstrate that *SFV* exhibits selective cytotoxicity, reduces clonogenicity and migration, and induces apoptosis. A conserved anticancer potential is highlighted by the convergence with research on other scorpion species. However, compensatory overexpression of anti-apoptotic proteins indicates adaptive resistance, which calls for more research to determine which peptides are active, how they interact with apoptotic regulators, and how well they work in conjunction with inhibitors or conventional treatments.

The NOD/SCID mouse strain was selected due to its immunodeficient background, which facilitates the establishment of human colorectal carcinoma xenografts while preserving tumor heterogeneity and metastatic potential [54]. In this study, DLD-1 cells implanted both orthotopically and subcutaneously successfully formed tumors, enabling evaluation of the biological effects of SFV in vivo. SFV treatment was associated with reduced tumor progression in both implantation models. The use of both orthotopic and subcutaneous xenograft models allowed complementary assessment of tumor behavior. Subcutaneous models enabled direct monitoring of tumor growth through inspection, palpation, and quantitative measurements, whereas orthotopic models more closely reflected the native colorectal tumor microenvironment. In orthotopic subgroups, *SFV* was administered IP, while in subcutaneous subgroups both intraperitoneal and intratumoral routes were employed, as intratumoral delivery has been reported to enhance local therapeutic effects [55]. The present study was not designed to compare *SFV* with standard chemotherapeutic agents; therefore, no positive chemotherapy control was included. Nevertheless, the observed antitumor effects are consistent with previous reports describing the anticancer potential of scorpion venoms. Fan et al. (2010) showed that intraperitoneal administration of *Mesobuthus martensii* Karsch venom suppressed glioblastoma progression and metastasis in rats, while Salem et al. (2016) demonstrated that *Androctonus amoreuxi* venom reduced solid tumor volume by 46.4% in a syngeneic mouse model [56, 57]. Similarly, in our study, tumors in the CDX group progressed steadily over 6 weeks, whereas tumor growth in the CDX + *SFV* group was markedly delayed. At week 6, mice in the orthotopic CDX + *SFV* subgroup exhibited preserved physiological functions including feeding, physical activity, and responsiveness, as well as normal behavioral parameters such as social interaction and facial grimace, similar to the control group. Post-mortem necropsy findings revealed widespread metastatic lesions in multiple organs in CDX mice, whereas only mild mesenteric cloudiness was detected in CDX + *SFV* mice. Additionally, histological examination reinforced the gross necropsy examinations, further confirming the pronounced reduction in primary tumor size in the CDX + *SFV* group compared with CDX alone. Taken together, these results suggest that *SFV* has significant antitumor potential, attenuating both tumor growth and metastatic spread. The observed preservation of physiological and behavioral functions further supports its therapeutic relevance, warranting additional mechanistic and translational studies.

The immunohistochemical analyses provided supportive evidence for the antitumor effects of *SFV* at the tissue level. The observed increase in Bax and p53 immunoreactivity, together with reduced Bcl-2 staining, is consistent with the involvement of mitochondrial apoptotic processes, in line with previous reports demonstrating that scorpion venom peptides can promote apoptosis through intrinsic pathways [40, 47]. In addition, altered KRAS and NF-κB immunoreactivity was observed in *SFV*-treated tumors. However, as immunohistochemistry provides semi-quantitative information, these changes should be interpreted as associative alterations in protein expression patterns rather than definitive evidence of direct signaling pathway inhibition. Accordingly, the observed changes do not distinguish between primary venom effects and secondary downstream responses. Taken together, the coexistence of apoptotic marker induction and altered oncogenic protein immunoreactivity may contribute to the phenotypic outcomes observed in vivo, including reduced tumor growth and metastatic spread. Importantly, these findings align with earlier studies reporting that scorpion venom components can influence angiogenesis and tumor invasion through modulation of integrin- and NF-κB-associated signaling networks [48]. Overall, our results support the antitumor activity of *SFV* at a functional and phenotypic level, including cytotoxic effects, apoptosis-associated responses, and suppression of tumor growth in xenograft models. Accordingly, the observed gene and protein expression changes should be interpreted as associative molecular responses rather than direct evidence of signaling pathway activation or inhibition. Elucidation of precise molecular targets and signaling mechanisms will require dedicated functional and pathway-specific investigations in future studies.

## Conclusion

This study demonstrated that *SFV* possesses potent and selective anticancer properties against colorectal carcinoma, providing a promising foundation for the development of novel therapeutic strategies. Comprehensive biochemical characterization revealed a complex proteomic profile enriched with low- and medium-molecular-weight peptides, many of which are known modulators of ion channels and apoptotic signaling pathways. Functional assays showed that *SFV* significantly inhibited viability, migration, and colony formation of CRC cell lines in a dose-dependent manner while sparing healthy colon fibroblasts, underscoring its therapeutic specificity. Importantly, flow cytometry and molecular analyses revealed that *SFV* triggered apoptosis through modulation of both intrinsic and extrinsic pathways, with upregulation of CASP8, BOK, and TNFRSF family members, as well as downregulation of oncogenic mediators such as BAK1 and TRAF3.

In vivo experiments provided further validation, as venom treatment suppressed tumor growth and reduced metastatic spread, with tumor volumes decreasing by approximately 30% and tumor weights reduced by more than 68% compared with untreated controls in orthotopic and subcutaneous xenograft mouse models. Histological and immunohistochemical analyses confirmed increased pro-apoptotic markers (Bax, p53) alongside decreased anti-apoptotic and oncogenic proteins (BcL-2, KRAS, NF-κB), suggesting that the venom not only induces apoptosis but also interferes with survival and proliferation signaling cascades. These findings highlight *SFV* as a natural source of bioactive compounds capable of targeting multiple hallmarks of colorectal cancer.

## Data Availability

The data supporting the findings of this study are available within the article. Additional data that support the findings of this study are available from the corresponding author upon reasonable request.
